# Targeting GPX4 and FSP1 to exploit ferroptotic vulnerability in Ewing sarcoma

**DOI:** 10.3389/fonc.2026.1945631

**Published:** 2026-09-28

**Authors:** Mian T. Mhindu, Stacia L. Koppenhafer, Mason G. Lyons, Jessica A.O. Zimmerman, David J. Gordon

**Affiliations:** 1 Department of Pediatrics, Division of Pediatric Hematology/Oncology, University of Iowa, Iowa City, IA, United States; 2 University of Iowa, Iowa City, IA, United States

**Keywords:** Ewing sarcoma, ferroptosis, FSP1, GPx4, ROS - reactive oxygen species

## Abstract

Ewing sarcoma tumors are defined by chromosomal translocations fusing *EWSR1* to ETS family genes, most commonly *FLI1*. The resulting EWS::FLI1 fusion oncoprotein drives tumorigenesis while simultaneously generating unique metabolic and molecular dependencies that may be exploited for Ewing sarcoma–specific therapies. Through analysis of the Cancer Dependency Map (DepMap, Broad Institute), we identified that Ewing sarcoma cell lines exhibit selective sensitivity to loss of genes regulating selenium metabolism compared to other cancer lineages. Ewing sarcoma cells are similarly sensitive to knockout of glutathione peroxidase 4 (GPX4), a selenoprotein that suppresses ferroptosis, a form of regulated, iron-dependent cell death characterized by the lethal accumulation of lipid peroxides. Using pharmacological inhibitors and targeted degraders of GPX4, as well as genetic knockout approaches, we demonstrate that Ewing sarcoma cells are selectively vulnerable to GPX4 loss or inhibition. We further identify ferroptosis suppressor protein 1 (FSP1) as a modulator of ferroptotic sensitivity in Ewing sarcoma, with FSP1 expression level serving as a determinant of the magnitude of ferroptotic response. Together, these findings establish ferroptosis as a targetable vulnerability in Ewing sarcoma, define GPX4 and FSP1 as key regulators of ferroptotic sensitivity in this disease, and provide a mechanistic rationale for therapeutic strategies targeting the ferroptosis defense axis in Ewing sarcoma tumors.

## Introduction

Ewing sarcoma is an aggressive bone and soft tissue malignancy that predominantly affects children, adolescents, and young adults, representing the second most common primary bone tumor in pediatric patients (1). Despite decades of research, the standard treatment for Ewing sarcoma, cytotoxic chemotherapy combined with surgery and/or radiation, has changed little, and outcomes for patients with metastatic or relapsed disease remain unacceptably poor (2). This underscores the urgent need to identify novel therapeutic vulnerabilities that can be exploited for more effective, disease-specific treatment strategies.

Ewing sarcoma is defined at the molecular level by chromosomal translocations that fuse the *EWSR1* gene to members of the ETS family of transcription factors, most commonly *FLI1*, giving rise to the EWS::FLI1 fusion oncoprotein (1). EWS::FLI1 functions as an aberrant transcription factor that drives oncogenesis through widespread dysregulation of gene expression, chromatin remodeling, and epigenetic reprogramming (1, 3, 4). While EWS::FLI1 itself has proven difficult to target pharmacologically, its expression generates a constellation of unique molecular and metabolic dependencies that distinguish Ewing sarcoma cells from normal tissues and other cancer types (5). This concept, that oncogene-driven tumorigenesis simultaneously creates selective vulnerabilities, has motivated systematic efforts to identify Ewing sarcoma–specific dependencies as candidates for therapeutic exploitation.

Ferroptosis is a form of regulated, non-apoptotic cell death driven by iron-dependent peroxidation of polyunsaturated fatty acid–containing phospholipids, resulting in the lethal accumulation of lipid peroxides (6–11). Unlike apoptosis, which is regulated primarily through caspase activation, ferroptosis is governed by a distinct set of metabolic and redox-regulatory pathways. The canonical suppressor of ferroptosis is glutathione peroxidase 4 (GPX4), a selenoprotein that reduces phospholipid hydroperoxides to less toxic lipid alcohols (12–14). GPX4 activity is dependent on glutathione as a cofactor, and its function is critically linked to selenium metabolism through the selenocysteine biosynthesis pathway (15). More recently, ferroptosis suppressor protein 1 (FSP1) has been identified as a parallel, GPX4-independent mechanism of ferroptosis resistance, functioning through reduction of ubiquinone (CoQ10) to ubiquinol, which acts as a lipophilic radical trap to quench lipid peroxide propagation (16–20). Together, GPX4 and FSP1 define two major axes of ferroptotic defense, and the relative contribution of each varies across cell types and contexts (11).

Ferroptosis has emerged as a therapeutically relevant vulnerability in multiple cancer types (6, 8, 11). Cancer cells frequently exhibit elevated iron uptake, altered lipid metabolism, and increased oxidative stress relative to normal tissues, creating a heightened dependence on ferroptosis defense mechanisms (6–8, 11). Pharmacological induction of ferroptosis using GPX4 inhibitors such as RSL3 and ML210, as well as targeted GPX4 degraders, has demonstrated preclinical efficacy across a range of solid tumors, and efforts to exploit ferroptotic sensitivity are increasingly recognized as a promising therapeutic strategy (11). Sensitivity to ferroptosis induction is shaped by multiple factors, including the expression levels of GPX4 and FSP1, cellular iron content, the composition of membrane phospholipids, and the availability of selenium and glutathione for GPX4 activity.

The relationship between EWS::FLI1-driven oncogenesis and ferroptotic vulnerability in Ewing sarcoma has not been systematically explored. However, several features of Ewing sarcoma biology suggest that ferroptosis may represent a particularly relevant vulnerability in this disease (21–26). EWS::FLI1 has been shown to reprogram metabolic and redox pathways, including those governing cysteine metabolism, glutathione synthesis, and lipid handling, all of which are central determinants of ferroptosis sensitivity (27–29). Several studies have demonstrated that Ewing sarcoma cell lines exhibit increased basal oxidative stress and dependence on antioxidant systems (27, 30–32). Recent work has also highlighted a functional link between polyamine metabolism and ferroptosis in Ewing sarcoma. Inhibition of ornithine decarboxylase with difluoromethylornithine (DFMO) reduces polyamine synthesis, leading to enhanced oxidative stress, depletion of cellular antioxidant capacity, and increased susceptibility to ferroptotic cell death (21). Collectively, these findings support a model in which EWS::FLI1-driven metabolic rewiring creates a ferroptosis-prone state that can be therapeutically exploited, while also suggesting that modulation of interconnected pathways, such as polyamine metabolism, may further sensitize Ewing sarcoma cells to ferroptotic induction.

In this study, we leveraged genome-wide CRISPR loss-of-function data from the Cancer Dependency Map (DepMap, Broad Institute) to perform an unbiased analysis of Ewing sarcoma–selective genetic dependencies (33). This analysis revealed a selective sensitivity of Ewing sarcoma cell lines to the loss of genes regulating selenium metabolism and, specifically, to the loss of the selenoprotein GPX4. We subsequently demonstrate, using pharmacological inhibitors, targeted GPX4 degraders, and genetic approaches, that Ewing sarcoma cells are selectively vulnerable to ferroptosis induction, and that FSP1 expression level is a determinant of the magnitude of ferroptotic response. These findings establish ferroptosis as a targetable vulnerability in Ewing sarcoma and define the GPX4/FSP1 axis as a regulator of ferroptotic sensitivity in this disease.

## Materials and methods

### Cell lines and culture

Cell lines were maintained at 37 °C in a humidified atmosphere containing 5% CO_2_. TC71 (RRID: CVCL_2213), EW8 (RRID: CVCL_1658), and A673 (RRID: CVCL_0800) cell lines were provided by Dr. Kimberly Stegmaier (Dana-Farber Cancer Institute, Boston, MA, USA). AGPN (RRID: CVCL_X981) cells were obtained from the Childhood Cancer Repository (Children’s Oncology Group). The ES6 (RRID: CVCL_1202) cell line was obtained from the Childhood Solid Tumor Network at St. Jude Children’s Research Hospital. HT1080 (RRID: CVCL_0317), U2OS (RRID: CVCL_0042), and BJ-fibroblast (RRID: CVCL_6573) cells were obtained from ATCC. Cells were cultured in Dulbecco’s Modified Eagle Medium (DMEM; Thermo Fisher Scientific) supplemented with 10% fetal bovine serum (FBS), 100 U/mL penicillin, and 100 μg/mL streptomycin. For non-adherent, spheroid culture, cells were grown in suspension in tissue culture plates coated with poly-HEMA. Cell line identity was confirmed by short tandem repeat (STR) profiling, and experiments were performed using cells within 8–10 passages following thawing. Cell lines were tested for mycoplasma contamination upon acquisition and approximately every six months thereafter using the Mycoplasma Detection Kit (Thermo Fisher Scientific, M7006).

### Chemical compounds

Ferrostatin-1, liproxstatin-1, RSL3, erastin, DC-2, ML-210, Z-VAD-FMK, and idebenone were obtained from MedChemExpress. Liproxstatin-1 and ferrostatin-1 were used at concentrations of 1 μM and 10 μM, respectively. Sodium selenite and poly-HEMA were obtained from Sigma-Aldrich. Doxycycline was obtained from ThermoFisher Scientific.

### GPX4-KO cell line

GPX4 knockout cell lines were generated using CRISPR/Cas9-mediated gene editing. Cells were transduced with a pLV[CRISPR] lentiviral vector (VectorBuilder) encoding Cas9, a GPX4-targeting guide RNA (5′-CACGCCCGATACGCTGAGTG-3′), and a puromycin resistance cassette. Forty-eight hours after transduction, cells were selected with puromycin and subsequently single-cell cloned by fluorescence-activated cell sorting using a FACS Aria Fusion cell sorter (BD Biosciences). Loss of GPX4 protein expression was confirmed by immunoblotting.

### Doxycycline-inducible expression of FSP1

The full-length FSP1 cDNA was obtained as a gene block (IDT; Coralville, IA) and cloned into a doxycycline inducible lentiviral vector (pLVX-TetOne-Puro; Takara Bio # 631849) as described in previous publications (30).

### Cell viability and drug dose response assays

Cell viability was assessed using the CellTiter-Glo Luminescent Cell Viability Assay (Promega, G9241) as previously described (30, 34). Briefly, 5,000–10,000 cells were seeded per well in 96-well plates and exposed to the indicated drug concentrations for 72 hours. CellTiter-Glo reagent was then added according to the manufacturer’s instructions, and luminescence was measured using a FLUOstar Omega microplate reader (BMG Labtech; RRID: SCR_025024). Half-maximal inhibitory concentration (IC_50_) values were calculated from log-transformed and normalized data using GraphPad Prism version 10.1.0 (RRID: SCR_002798).

### Detection of lipid peroxidation

Lipid peroxidation was measured using the BOPIDY 581/591 C11 lipid peroxidation sensor (Thermo Fisher Scientific) according to the manufacturer’s instructions. Briefly, cells were seeded in 6-well plates and treated as indicated in the figure legends. Following treatment, cells were incubated with 2 μM BODIPY 581/591 C11 in complete culture medium for 30 minutes at 37 °C in a humidified incubator containing 5% CO_2_. Cells were then washed twice with phosphate-buffered saline (PBS), harvested by trypsinization, and resuspended in ice-cold PBS containing 2% fetal bovine serum. Fluorescence was analyzed by flow cytometry using a LSR II cytometer (Becton Dickson). Data were analyzed using FlowJo software (BD Biosciences; RRID: SCR_008520).

### Protein isolation and immunoblotting

Whole-cell protein lysates were prepared by incubating cells in RIPA buffer (Boston BioProducts) supplemented with Halt Protease and Phosphatase Inhibitor Cocktail, EDTA-free (Thermo Fisher Scientific), for 20 minutes on ice. Lysates were clarified by centrifugation at 17,000 × g for 20 minutes at 4 °C, and supernatants were collected for analysis. Proteins were separated by SDS-PAGE and transferred to polyvinylidene difluoride (PVDF) membranes (Millipore). Membranes were sectioned using a pre-stained molecular weight ladder (Thermo Fisher Scientific) as a guide prior to antibody incubation. Primary antibodies against GPX4 (Proteintech, #67763; 1:1000; AB_2919543), FSP1 (Proteintech, #20886, 1:1000; AB_2878756), and vinculin (Proteintech, #26520; 1:5000; RRID: AB_2919877) were used. Immunoblot band intensities were quantified using Fiji software (RRID: SCR_002285).

### Cancer dependency map

Gene expression and CRISPR dependency data from the Cancer Dependency Map resource were accessed on September 18, 2025 through the DepMap Portal (depmap.org; RRID: SCR_017655) (33). Gene dependencies enriched for Ewing sarcoma tumors versus all other CRISPR screened tumors were identified using a two-sided T-test on the Chronos CRISPR Gene Effect scores. Gene effect difference was calculated as a T-statistic score by the DepMap Portal. P-values were corrected for multiple hypothesis testing using the Benjamini-Hochberg procedure (FDR<0.1). Gene set enrichment analysis was performed using ShinyGo 0.85.1 (RRID: SCR_019213) (35–37).

### Statistical analysis

Statistical analyses were performed using GraphPad Prism version 11 (RRID: SCR_002798). Data are presented as mean values with individual biological replicates shown when applicable. Comparisons between two groups were performed using unpaired two-tailed Student’s *t*-tests. Comparisons among three or more groups were performed using one-way analysis of variance (ANOVA) followed by Tukey’s multiple-comparisons test. A *P* value ≤ 0.05 was considered statistically significant.

## Results

### Selenium metabolism and GPX4 are unique dependencies in Ewing sarcoma

To identify unique dependencies of Ewing sarcoma tumors, we analyzed gene knockout data from the Cancer Dependency Map (DepMap, Broad Institute) (33, 38). A total of 91 genes demonstrated selective toxicity (FDR<0.1) in Ewing sarcoma relative to other tumor types (**Supplementary Table 1**). Genes with well-established, essential roles in Ewing sarcoma biology, including *EWSR1*, *FLI1*, *CDK4*, *TRIM8*, and *STAG1*, ranked among the top hits for selective toxicity, validating the screening approach (39–44). We then applied gene set enrichment analysis (GSEA) to identify pathways overrepresented among these 91 genes (35). The top-ranked gene set was selenocompound metabolism, driven by *SEPHS2*, *SEPSECS*, *PSTK*, and *TXNRD1* (**Figure 1A**; **Supplementary Figure 1**) (45). We also identified *GPX4*, a selenoprotein and core suppressor of ferroptosis, as a top selective dependency, with an effect size comparable to that of *EWSR1* and *FLI1* (**Figures 1B, C**) (15). Notably, this dependency was specific to *GPX4* and was not shared by other members of the glutathione peroxidase family (*GPX1–3*, *GPX5–8*), suggesting a non-redundant and functionally distinct role for GPX4 in Ewing sarcoma survival (**Figure 1D**).

**Figure 1:**
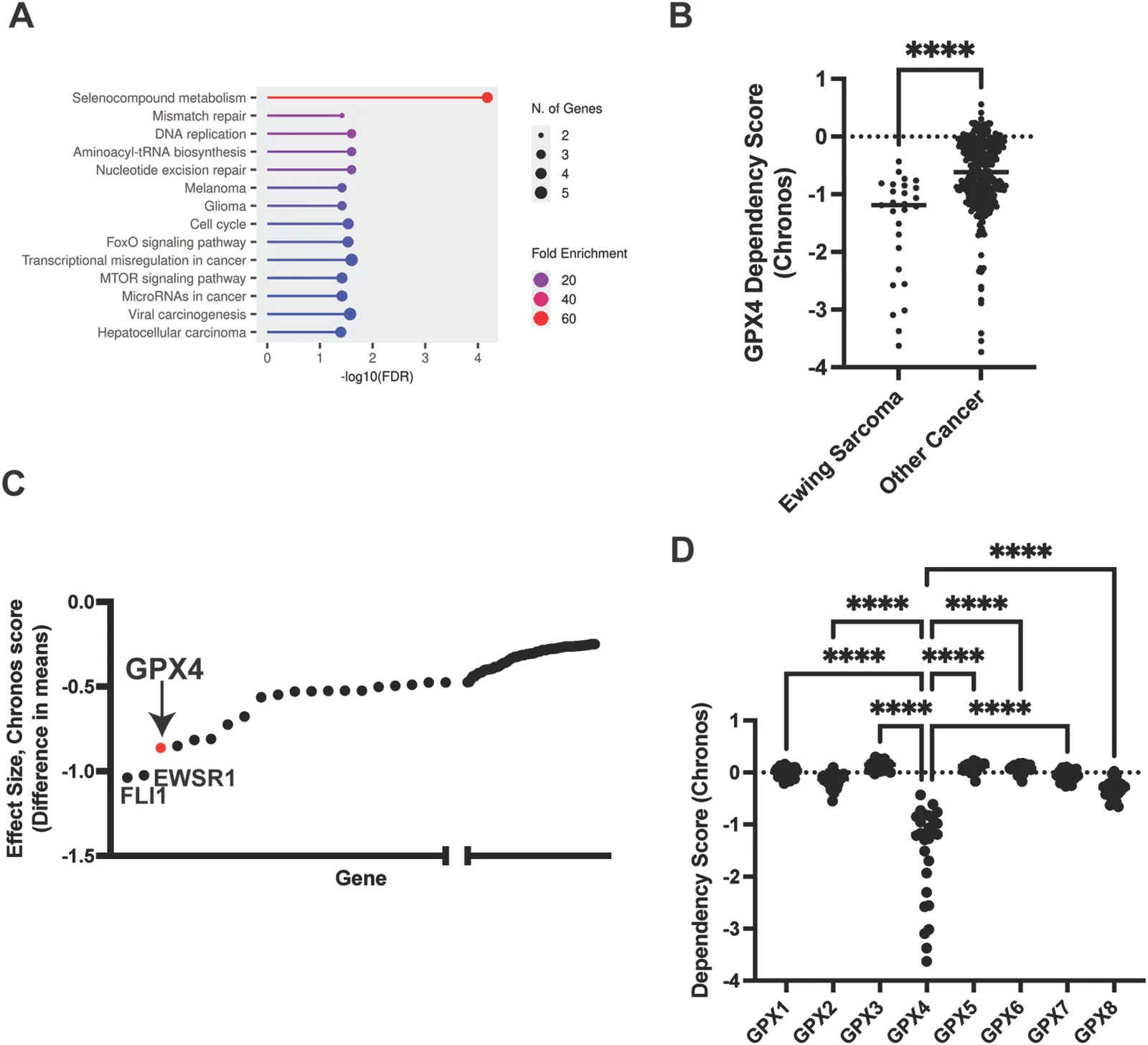
Selenium metabolism and GPX4 are unique dependencies in Ewing sarcoma. **(A)** Gene set enrichment analysis of genes that demonstrate selective toxicity in Ewing sarcoma relative to other tumor types. **(B)** CRISPR gene knockout dependency score for GPX4. **(C)** Effect size of top-ranked genes that demonstrate selective toxicity for Ewing sarcoma cell lines relative to other tumor types. **(D)** CRISPR dependency score for GPX1–8 for Ewing sarcoma cell lines. Dependency data are from DepMap (depmap.org) ****, P < 0.0001.

### Knockout of GPX4 in Ewing sarcoma cells induces ferroptosis

Next, we used CRISPR/Cas9 to generate GPX4 knockout (GPX4-KO) cells in the EW8 Ewing sarcoma cell line. Anticipating that loss of GPX4 would be lethal or highly toxic, gene editing was performed in the presence of ferrostatin-1, a potent ferroptosis inhibitor that rescues cell death induced by GPX4 loss or inhibition (**Figure 2A**) (10, 38). Multiple single-cell–derived clones were established, and representative data from two independent clones are shown. Short-term withdrawal of ferrostatin-1 (4 hours) led to a marked increase in oxidation of C11-BODIPY, a sensitive fluorescent probe of lipid peroxidation (**Figure 2B**) (39, 40). Consistent with this, removal of ferrostatin-1 resulted in near-complete loss of cell viability (**Figure 2C**). Because cell–cell interactions and growth conditions (adherent versus suspension) can influence ferroptosis sensitivity, we next examined GPX4-KO cells grown as spheroids in non-adherent conditions (41). Under these conditions, withdrawal of ferrostatin-1 similarly resulted in rapid loss of viability (**Figure 2D**). To confirm that this dependency was not cell line–specific, we generated GPX4-KO cells in a second Ewing sarcoma cell line, TC71, under ferrostatin-1 rescue conditions. Consistent with findings in EW8 cells, removal of ferrostatin-1 in TC71 GPX4-KO cells was also lethal (**Figures 2E, F**; **Supplementary Figure 2**).

**Figure 2:**
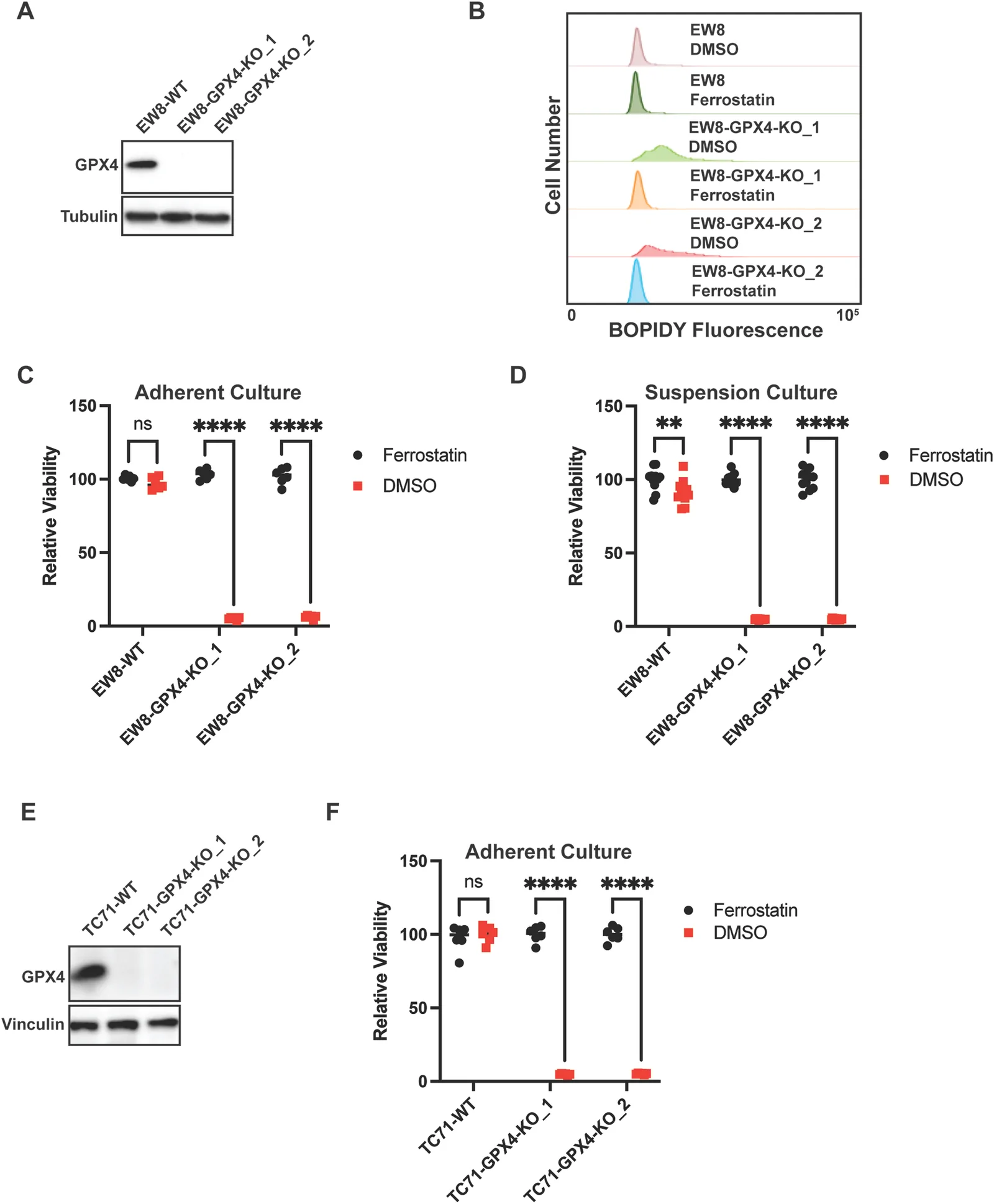
Knockout of GPX4 in Ewing sarcoma cells induces ferroptosis. **(A)** GPX4 expression in EW8-GPX4-KO cells grown in the presence of ferrostatin. **(B)** EW8-GPX4-KO cells were grown with ferrostatin or DMSO and then lipid peroxidation was quantified using C11-BOPIDY fluorescence. **(C)** EW8-GPX4-KO cells were grown with ferrostatin or DMSO for 72 hours and then cell viability was quantified using Cell-Titer-Glo, n=6 replicates per condition. **(D)** EW8-GPX4-KO cells were grown in suspension as spheroids with ferrostatin or DMSO for 72 hours. Cell viability was quantified using Cell-Titer-Glo, n=6 replicates per condition. **(E)** GPX4 protein expression in TC71-GPX4-KO cells grown in the presence of ferrostatin. **(F)** TC71-GPX4-KO cells were grown in ferrostatin or DMSO for 72 hours and then cell viability was quantified using Cell-Titer-Glo, n=6 replicates per condition **, P < 0.01, ****, P < 0.0001.

### Targeting GPX4 with a small-molecule degrader recapitulates effects of GPX4 knockout

To complement these genetic studies, we employed the small-molecule GPX4 degrader DC-2 to reduce GPX4 protein levels in Ewing sarcoma cell lines (**Figure 3A**) (42). GPX4 degradation led to increased oxidation of C11-BODIPY, a fluorescent sensor of lipid peroxidation (**Figure 3B**), and significantly decreased cell viability in both EW8 and TC71 cells (**Figures 3C, D**).

**Figure 3:**
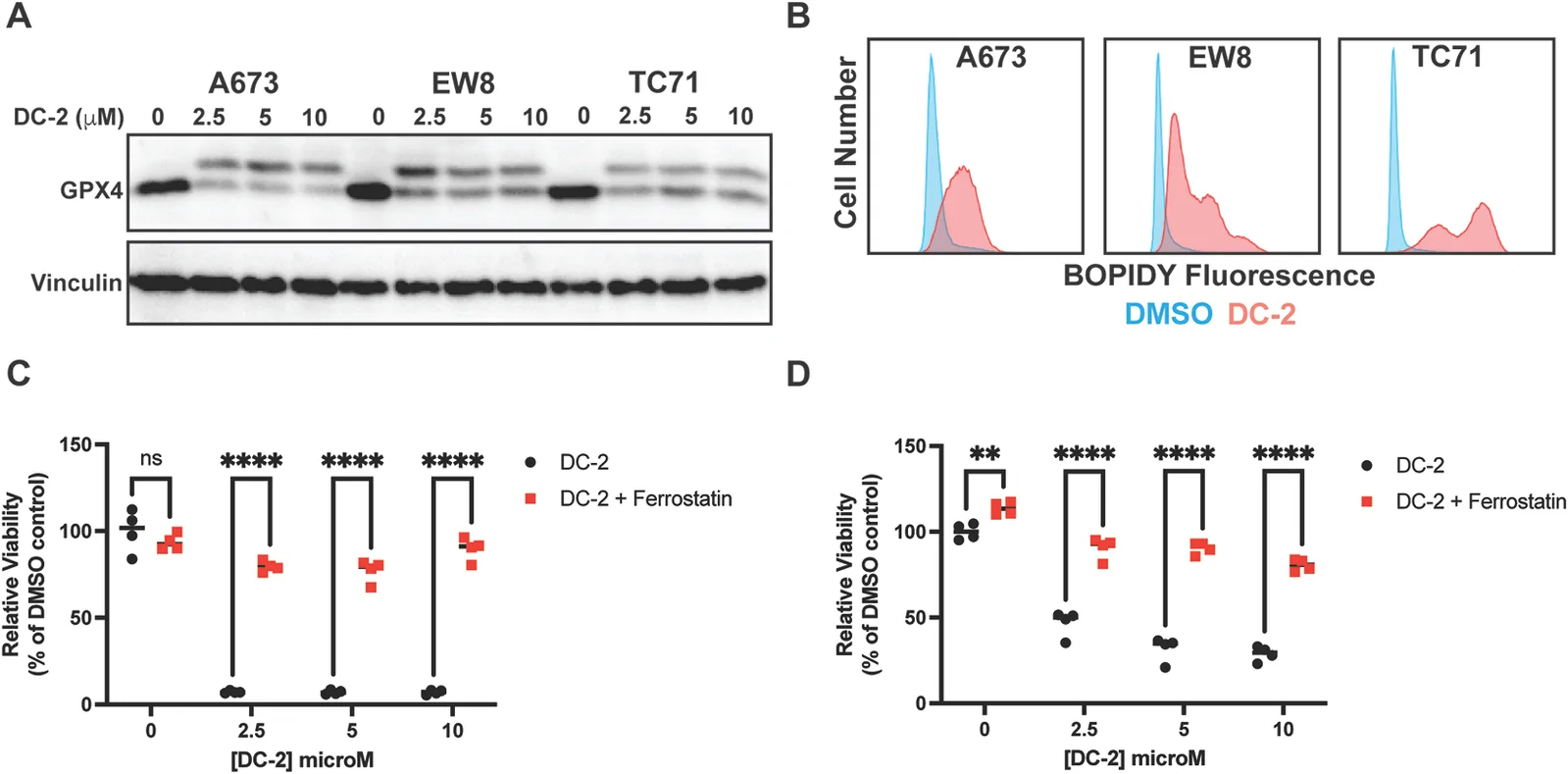
Targeting GPX4 with a small-molecule degrader. **(A)** Ewing sarcoma cells were treated with DC-2 for 24 hours. **(B)** Ewing sarcoma cells were treated with DMSO or DC-2 (2.5 μM) and then lipid peroxidation was quantified using C11-BOPIDY fluorescence. **(C, D)** EW8 **(C)** and TC71 **(D)** cells were treated with different doses of DC-2, in the presence or absence of ferrostatin-1, for 72 hours and then cell viability was quantified using Cell-Titer-Glo, n=4 replicates **, P < 0.01 ****, P < 0.0001.

### Inhibition of GPX4 in Ewing sarcoma induces ferroptosis

Consistent with these findings, Ewing sarcoma cell lines were highly sensitive to RSL3, a potent covalent inhibitor of GPX4 (**Figure 4A**) (38, 43). Notably, their sensitivity was comparable to that of the fibrosarcoma HT1080 cell line, a well-established ferroptosis-sensitive model, and greater than that observed in the relatively resistant osteosarcoma U2OS cell line (10). RSL3 treatment also increased C11-BODIPY oxidation, indicating enhanced lipid peroxidation (**Figure 4B**). Importantly, the ferroptosis inhibitors ferrostatin-1 and liproxstatin-1 both rescued RSL3-induced cytotoxicity across multiple Ewing sarcoma cell lines (**Figures 4C, D**) (44). In contrast, the pan-caspase inhibitor Z-VAD-FMK did not rescue the effect of RSL3 on Ewing sarcoma cells (**Supplementary Figure 3**). Treatment with a structurally distinct GPX4 inhibitor, ML-210, produced similar results (**Figure 4E**) (42). Because GPX4 requires glutathione (GSH) as a cofactor, we further assessed the effects of erastin, which depletes GSH by inhibiting the system Xc− cystine transporter (10). Erastin treatment likewise reduced cell viability and this effect was rescued by ferrostatin-1, consistent with induction of ferroptosis (**Figure 4F**).

**Figure 4:**
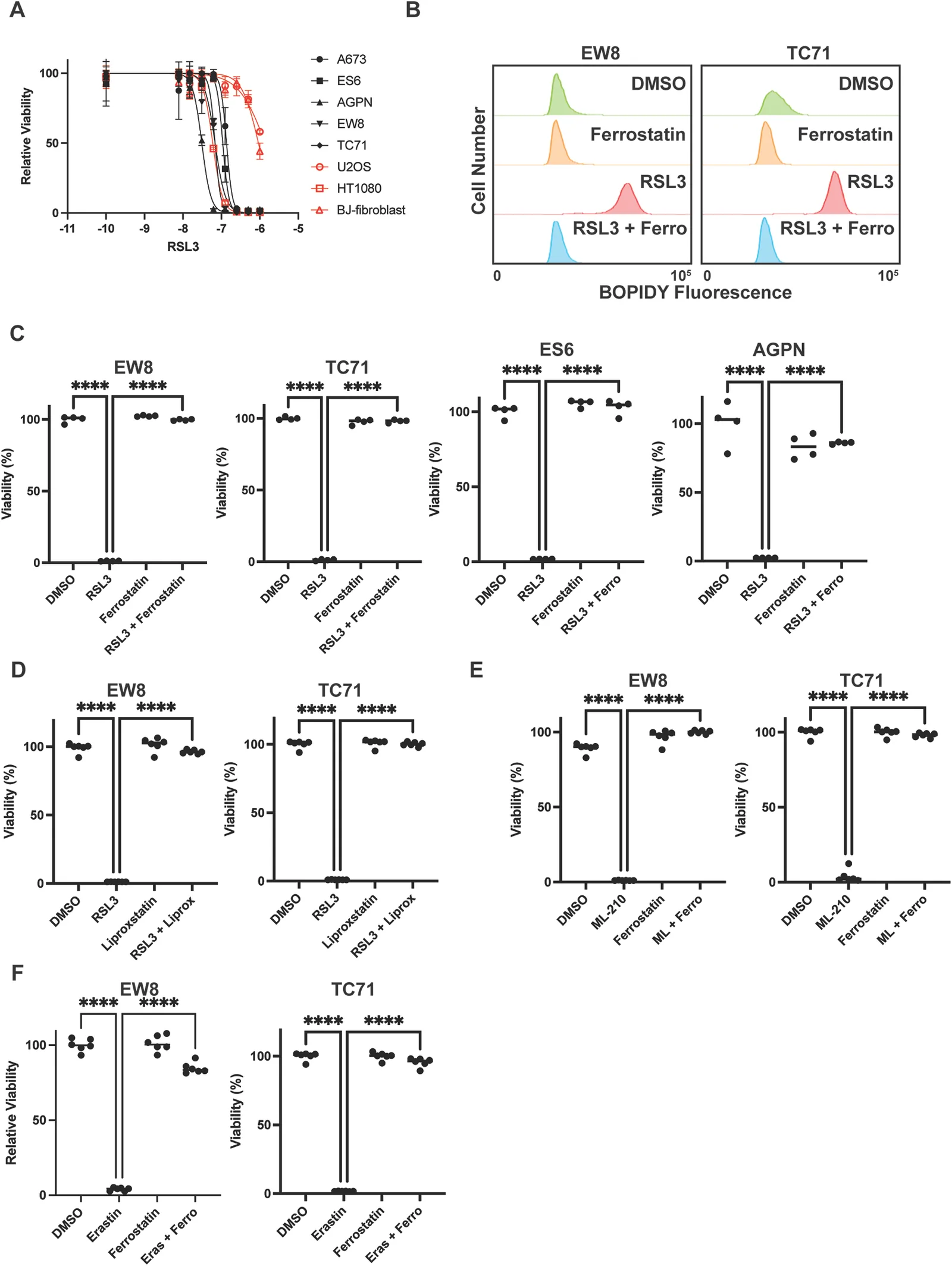
Inhibition of GPX4 in Ewing sarcoma induces ferroptosis. **(A)** Dose response curves for cell lines treated with RSL3. Ewing sarcoma cell lines are in black and other cell lines are in red. Cell viability was assessed 72 hours after drug was added using Cell-Titer-Glo. Error bars represent the mean ± SD of four replicates. The results are representative of two independent experiments. **(B)** EW8 and TC71 cells were treated with RSL3 (500 nM), in the presence or absence of ferrostatin, and then lipid peroxidation was quantified using C11-BOPIDY fluorescence. **(C)** Ewing sarcoma cell lines were treated with RSL3, in the presence or absence of ferrostatin, for 72 hours and then cell viability was quantified using Cell-Titer-Glo, n=4 replicates. **(D)** Ewing sarcoma cell lines were treated with RSL3 (500 nM), in the presence or absence of liproxstatin, for 72 hours and then cell viability was quantified using Cell-Titer-Glo, n=4 replicates. **(E)** Ewing sarcoma cell lines were treated with ML-210 (500 nM), in the presence or absence of ferrostatin, for 72 hours and then cell viability was quantified using Cell-Titer-Glo, n=4 replicates. **(F)** Ewing sarcoma cell lines were treated with erastin (500 nM), in the presence or absence of ferrostatin, for 72 hours and then cell viability was quantified using Cell-Titer-Glo, n=4 replicates ****, P < 0.0001.

### FSP1 suppresses ferroptosis in Ewing sarcoma cells

Ferroptosis suppressor protein 1 (FSP1) is a GPX4-independent ferroptosis suppressor that functions via a CoQ10-dependent radical-trapping mechanism (17, 18). FSP1 catalyzes reduction of ubiquinone (CoQ10) to ubiquinol, which acts as a lipophilic radical trap to quench lipid peroxide propagation (18). Loss of FSP1 alone is not typically sufficient to induce ferroptosis but can sensitize cells to other ferroptosis inducers (16, 19, 20, 53). Consistent with this, Ewing sarcoma cell lines express lower levels of *FSP1* mRNA than other cancer cell types (DepMap, Broad Institute), yet FSP1 knockout is not toxic (**Figures 5A, B**). Supporting a functional role for the FSP1/CoQ10 axis in Ewing sarcoma, idebenone—a synthetic, more water-soluble ubiquinol analog—reduced RSL3 toxicity in multiple Ewing sarcoma cell lines (**Figures 5C–E**) (45). To further interrogate FSP1 function, we used a doxycycline-inducible system (TO-FSP1) to overexpress FSP1 and found that increasing FSP1 expression was sufficient to decrease sensitivity to RSL3 (**Figures 5F–H**).

**Figure 5:**
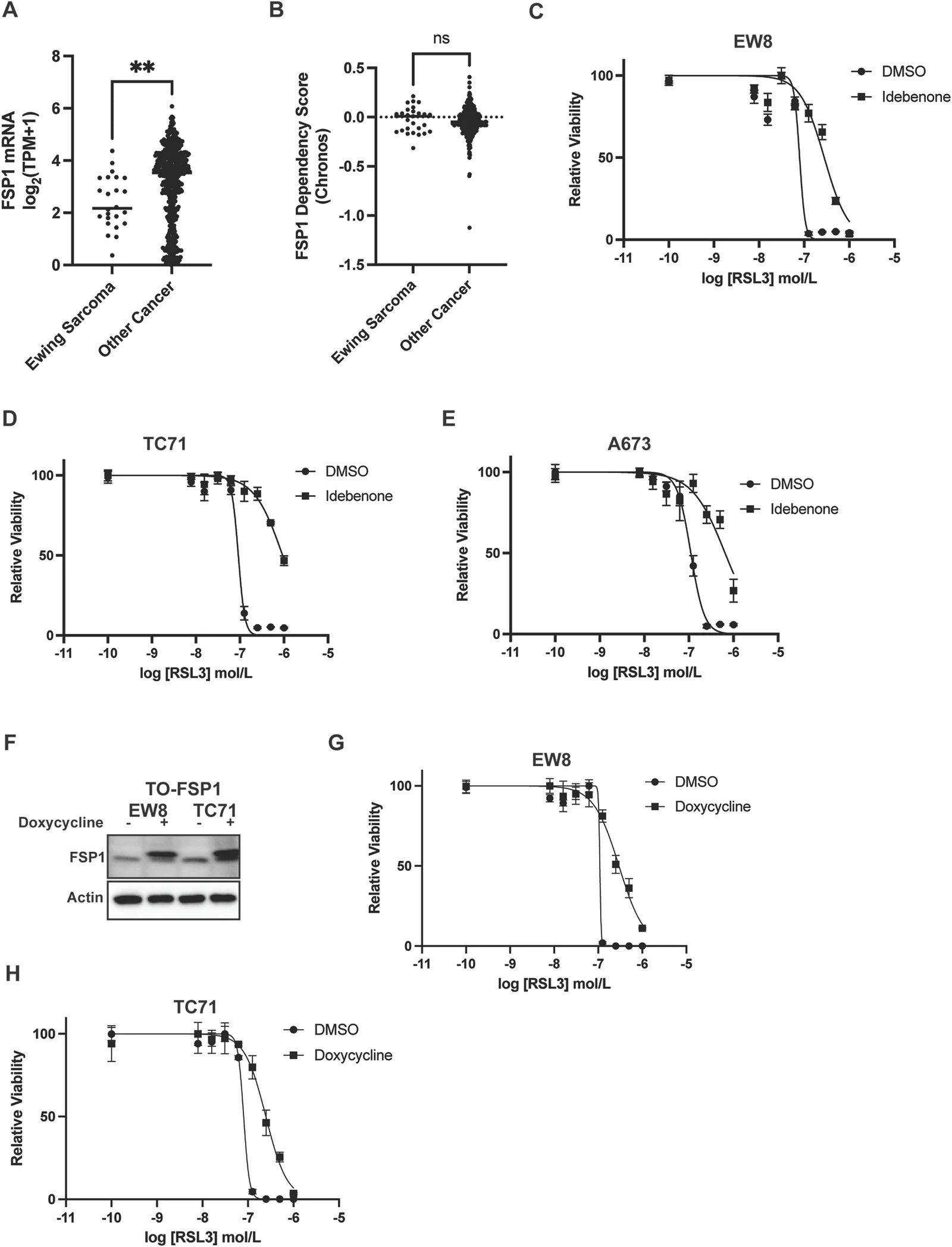
FSP1 suppresses ferroptosis in Ewing sarcoma cells. **(A)** Expression of FSP1 mRNA in cancer cell lines (depmap.org). **(B)** CRISPR gene knockout dependency score for FSP1 (depmap.org). **(C–E)** Dose response curves for Ewing sarcoma cell lines treated with RSL3, in the presence or absence of idebenone (10 μM). Cell viability was assessed 72 hours after drug was added using Cell-Titer-Glo. Error bars represent the mean ± SD of four replicates. **(F)** EW8 and TC71 cells with doxycycline-inducible expression of FSP1 were treated with DMSO or doxycycline (1 μM) for 24 hours and then cellular lysates were collected for immunoblotting. **(G, H)** EW8 and TC71 cells with doxycycline-inducible expression of FSP1 were treated with DMSO or doxycycline (1 μM) and different doses of RSL3. Cell viability was assessed 72 hours after drug was added using Cell-Titer-Glo. Error bars represent the mean ± SD of four replicates **, P < 0.01.

### Selenium regulates the level of GPX4 in Ewing sarcoma cells

In contrast to FSP1, Ewing sarcoma cell lines express *GPX4* mRNA at levels comparable to those observed in other cancer types (**Figure 6A**) (33). However, selenium availability is not standardized across cell culture conditions and may influence expression of the GPX4 selenoprotein (46–48). In this study, EW8 and TC71 cells were cultured in standard DMEM (Dulbecco’s Modified Eagle Medium), which lacks selenium, supplemented with fetal bovine serum (FBS) as a variable selenium source (49). Under these conditions, selenium availability may be limiting. To test whether selenium restricts GPX4 synthesis, cells were cultured in standard media with supplementation of sodium selenite. Selenium supplementation increased GPX4 protein levels 1.3–1.4-fold (**Figure 6B**) and was sufficient to confer modestly increased resistance to the GPX4 inhibitor RSL3 (**Figures 6C, D**). These findings demonstrate that selenium availability directly affects GPX4 protein abundance and can alter responses to pharmacologic GPX4 inhibition. Thus, while genetic knockout studies establish GPX4 as an essential dependency in Ewing sarcoma, the magnitude of sensitivity to GPX4-targeted therapies may vary depending on selenium levels in the cellular environment. This observation may help explain differences in ferroptosis susceptibility reported across experimental systems and underscores the importance of carefully considering selenium content and other micronutrients when interpreting studies of GPX4 dependence and ferroptosis in cancer cells.

**Figure 6:**
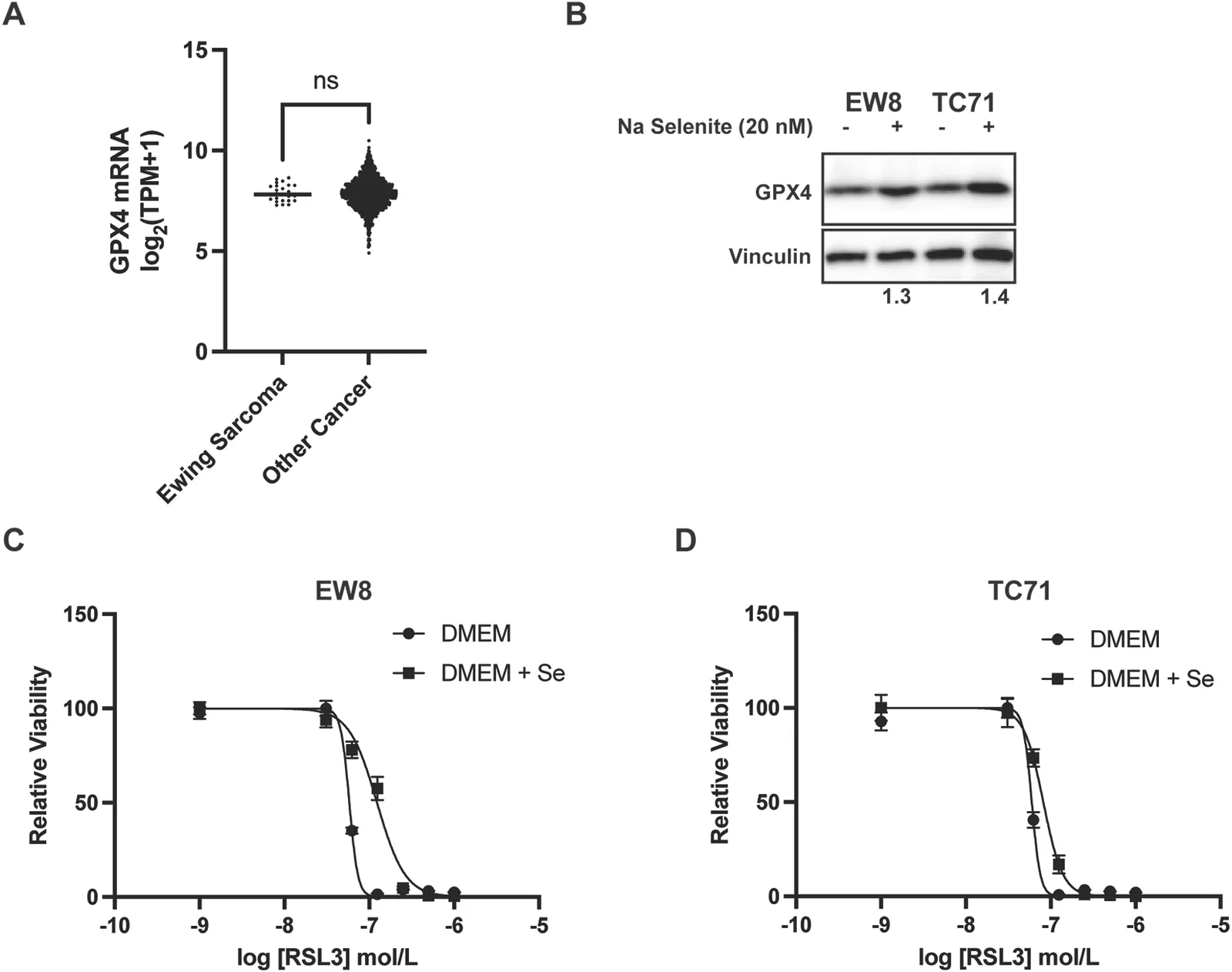
Selenium regulates the level of GPX4 in Ewing sarcoma cells. **(A)** Expression of GPX4 mRNA in cancer cell lines (depmap.org). **(B)** Ewing sarcoma cells were grown in standard media or media supplemented with sodium selenite (20 nM). Relative GPX4 expression in selenium-treated and control-treated cells, normalized to the vinculin loading control, is shown. **(C, D)** Dose response curves for Ewing sarcoma cell lines treated with RSL3, with or without sodium selenium supplementation (20 nM). Cell viability was assessed 72 hours after drug was added using Cell-Titer-Glo. Error bars represent the mean ± SD of four replicates.

## Discussion

In this work, we identify ferroptosis as a targetable vulnerability in Ewing sarcoma and define the GPX4/FSP1 axis as a central determinant of ferroptotic sensitivity in this disease. Through analysis of CRISPR loss-of-function data from the Cancer Dependency Map, we found that Ewing sarcoma cell lines exhibit a striking and selective dependency on GPX4, with an effect size comparable to the cancer-defining oncogenic drivers *EWS::FLI1*, *CDK4*, *TRIM8*, and *STAG1* (33). Our genetic and pharmacologic experiments demonstrate that GPX4 suppression, via CRISPR knockout, targeted degradation, or small-molecule inhibition, results in rapid lipid peroxidation followed by cell death. We were unable to use iron chelators to demonstrate iron-dependent cell death, a feature of ferroptosis, because Ewing sarcoma cells are known to be highly sensitive to iron chelation, precluding clear interpretation of rescue experiments (50–52). However, the observation that ferroptosis inhibitors, including ferrostatin-1 and liproxstatin-1, fully rescue cell viability support ferroptosis as the mechanism of cell death. Overall, these findings demonstrate that GPX4 is critical for Ewing sarcoma cell survival and support ferroptotic sensitivity as a metabolic liability.

One notable feature of our findings is that Ewing sarcoma cell lines exhibit low basal expression of FSP1, a GPX4-independent ferroptosis suppressor that protects cells through a CoQ10-dependent radical-trapping mechanism (18). Loss of FSP1 alone is generally insufficient to induce ferroptosis but can sensitize cells to ferroptosis-inducing agents (16, 19, 20, 53). Overexpression of FSP1 in Ewing sarcoma cells was sufficient to increase resistance to the GPX4 inhibitor RSL3 and idebenone, a synthetic ubiquinol analog, similarly attenuated ferroptotic cell death. These results highlight the impact of the FSP1/CoQ10 pathway in shaping the ferroptotic threshold of Ewing sarcoma cells. More broadly, they suggest that the endogenous level of FSP1 may contribute, among other factors, to the GPX4 dependency observed in this tumor type.

Our results also raise important considerations regarding selenium availability and GPX4 expression. Because GPX4 is a selenoprotein whose synthesis requires sufficient selenium, standard culture conditions, particularly formulations that lack defined selenium content, may influence GPX4 abundance (47–49). Selenium supplementation increased GPX4 protein levels and reduced sensitivity to RSL3, suggesting that selenium limitation may further exacerbate ferroptotic vulnerability (49). While this observation does not diminish the relevance of GPX4 dependency in Ewing sarcoma, it highlights the need to consider selenium availability when interpreting *in vitro* ferroptosis studies and suggests that selenium metabolism itself may represent a regulatory node influencing GPX4 activity *in vivo*.

An important question raised by our findings is how GPX4 inhibition might be advanced toward clinical therapies (54–57). Although covalent GPX4 inhibitors exhibit potent preclinical activity, their translation has been hindered by off-target protein interactions, toxicity, and poor metabolic stability (44). These pharmacologic challenges have motivated alternative approaches such as GPX4-targeted protein degraders, which bypass the need for covalent inhibition and have shown enhanced potency in experimental systems. However, achieving adequate systemic exposure, tumor selectivity, and favorable safety profiles remains a major obstacle (54). Parallel strategies, including inhibition of upstream pathways governing cystine uptake, glutathione synthesis, or selenium metabolism, may offer approaches for indirectly suppressing GPX4 activity (57). Together, these ongoing efforts highlight both the opportunities and challenges of clinically targeting GPX4 and suggest that Ewing sarcoma, due to its dependence on GPX4, may represent a particularly compelling tumor type for evaluation of GPX4-directed therapies.

The mechanistic basis for heightened ferroptotic susceptibility in Ewing sarcoma is likely multifactorial. Prior studies demonstrate that the EWS::FLI1 fusion oncoprotein rewires redox metabolism, alters cysteine and glutathione homeostasis, and promotes high basal oxidative stress, all factors that increase reliance on lipid peroxide detoxification (27–29). Moreover, links between polyamine metabolism and ferroptosis, along with evidence of impaired antioxidant buffering capacity, further support the concept that Ewing sarcoma cells exist in a ferroptosis-prone state (21). Our results extend this framework by demonstrating that disruption of either major ferroptosis defense arm, GPX4-dependent reduction of lipid hydroperoxides or FSP1-dependent CoQ10 recycling, alters cell fate in this cancer.

This study has several limitations. First, all experiments were performed in established Ewing sarcoma cell lines cultured *in vitro*, and the relevance of GPX4/FSP1-dependent ferroptotic vulnerability was not validated *in vivo* using xenograft or patient-derived models. Consequently, the impact of tumor microenvironmental factors, such as iron availability and immune cell interactions, on ferroptotic sensitivity are untested. Second, core functional studies, including GPX4 knockout, degrader treatment, and FSP1 overexpression, were largely confined to two cell lines, EW8 and TC71. Although the DepMap dependency data and select pharmacologic experiments included additional cell lines, broader validation across a larger and more molecularly diverse panel would strengthen generalizability. Third, the pharmacologic tools used to induce ferroptosis, including RSL3, ML-210, and DC-2, are established research compounds rather than clinically viable therapeutics. Because no clinical grade GPX4-targeted agent was investigated, due to the drug limitations discussed above, the translational potential of this vulnerability remains to be established. Fourth, the finding that selenium supplementation modestly increased GPX4 protein levels and RSL3 resistance highlights that selenium and other micronutrient content in culture media could introduce a potential source of variability when comparing results with other published work. Finally, while EWS::FLI1-driven metabolic rewiring is proposed as the mechanistic basis for heightened ferroptotic susceptibility, this study did not directly test whether EWS::FLI1 expression level or activity causally determines the magnitude of GPX4 dependency, leaving the precise molecular link between the fusion oncoprotein and ferroptotic vulnerability to be defined in future work.

In summary, our study establishes ferroptosis as an exploitable vulnerability in Ewing sarcoma. By defining the essential role of GPX4 and the modulatory influence of FSP1, we provide a mechanistic foundation for therapeutic strategies aimed at leveraging ferroptotic cell death in this aggressive pediatric cancer. Future work will be directed toward defining the relevance of ferroptosis *in vivo*, exploring how ferroptotic stress interfaces with the tumor microenvironment, and developing clinically viable approaches to target the GPX4/FSP1 ferroptosis defense axis in Ewing sarcoma.

## Data Availability

The original contributions presented in the study are included in the article/**Supplementary Material**. Further inquiries can be directed to the corresponding author.

## Supplementary material

Supplementary Figure 1 Selenocompound metabolism genes (KEGG graph; gene set enrichment analysis) that demonstrate selective toxicity in Ewing sarcoma relative to other tumor types.

Supplementary Figure 2 TC71-GPX4-KO cells were grown in suspension as spheroids with ferrostatin or DMSO for 72 hours. Cell viability was quantified using Cell-Titer-Glo, n=6 replicates per condition.

Supplementary Figure 3 EW8 and TC71 cells were treated with RSL3 (500 nM), Z-VAD-FMK (10 μM), or the drug combination for 72 hours. Cell viability was then assessed using Cell-Titer-Glo, n=6 replicates.

Supplementary Table 1 Gene knockouts exhibiting selective toxicity in Ewing sarcoma compared with other tumor types, identified by CRISPR dependency analysis (depmap.org).
